# Dissolving Candlelit Microneedle for Chronic Inflammatory Skin Diseases

**DOI:** 10.1002/advs.202004873

**Published:** 2021-05-07

**Authors:** Jungyoon Ohn, Mingyu Jang, Bo Mi Kang, Huisuk Yang, Jin Tae Hong, Kyu Han Kim, Ohsang Kwon, Hyungil Jung

**Affiliations:** ^1^ Department of Dermatology Seoul National University College of Medicine Seoul 03080 Republic of Korea; ^2^ Institute of Human‐Environment Interface Biology Medical Research Center Seoul National University Seoul 03080 Republic of Korea; ^3^ Laboratory of Cutaneous Aging and Hair Research Biomedical Research Institute Seoul National University Hospital Seoul 03080 Republic of Korea; ^4^ Department of Biotechnology Yonsei University 50 Yonsei‐ro Seoul 03722 Republic of Korea; ^5^ JUVIC Inc. 272 Digital‐ro Seoul 08389 Republic of Korea

**Keywords:** candlelit microneedles, chronic inflammatory skin diseases, dissolving microneedles, triamcinolone acetonide

## Abstract

Chronic inflammatory skin diseases (CISDs) negatively impact a large number of patients. Injection of triamcinolone acetonide (TA), an anti‐inflammatory steroid drug, directly into the dermis of diseased skin using needle‐syringe systems is a long‐established procedure for treating recalcitrant lichenified lesions of CISDs, referred to as TA intralesional injection (TAILI). However, TAILI causes severe pain, causing patients to be stressed and reluctant to undergo treatment. Furthermore, the practitioner dependency on the amount and depth of the injected TA makes it difficult to predict the prognosis. Here, candle flame (“candlelit”)‐shaped TA‐loaded dissolving microneedles (Candlelit‐DMN) are designed and fabricated out of biocompatible and biodegradable molecules. Candlelit‐DMN distributes TA evenly across human skin tissue. Conjoined with the applicator, Candlelit‐DMN is efficiently inserted into human skin in a standardized manner, enabling TA to be delivered within the target layer. In an in vivo skin inflammation mouse model, Candlelit‐DMN inserted with the applicator effectively alleviates inflammation by suppressing inflammatory cell infiltration and cytokine gene expression, to the same extent as TAILI. This Candlelit‐DMN with the applicator arouses the interest of dermatologists, who prefer it to the current TAILI procedure.

## Graphical abstract

Candle flame‐shaped triamcinolone acetonide‐loaded dissolving microneedle is designed and fabricated, out of biocompatible molecules. With an applicator, the microneedles are efficiently inserted into human skin in a standardized manner without pain, enabling the drug to be delivered within the target layer. In an in vivo skin inflammation mouse model, the microneedles inserted with the applicator effectively alleviate inflammation.

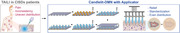

## 1 Introduction

**Figure 1:**
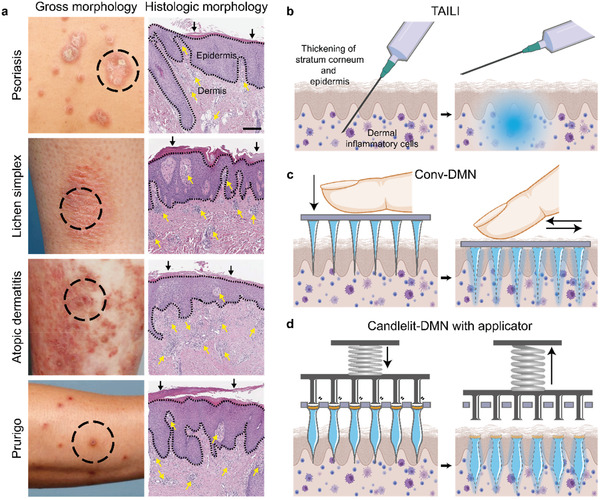
Clinical and histological findings of CISDs and schematics of the current TAILI procedure and DMN application. a) Clinical and histological findings of four representative CISDs. Dense dermal inflammatory cell infiltration (yellow arrow) with hypertrophied stratum corneum (black arrow) and/or thickened epidermis with inflammation (dotted black line) are present. Black bar: 200 µm. b) Schematic diagram of the TAILI procedure for CISDs, mainly targeting the upper dermis and lower epidermis. c) Conv‐DMN applied by finger. d) Candlelit‐DMN with applicator.

Chronic inflammatory skin diseases (CISDs), such as psoriasis, atopic dermatitis, or eczematous pruritic disease, continue to distress a large number of patients worldwide and cause disability in daily life.^[^ ^1^ ^]^ The global prevalence of psoriasis is 0.14 to 1.99%,^[^ ^2^ ^]^ and atopic dermatitis, a representative chronic eczematous disease, affects 10 to 30% of the general population.^[^ ^3^ ^]^ The chronic and relapsing features of the diseases establish scaly lichenified lesions on the skin (**Figure** **1**a), substantially impairing patients’ and their families’ quality of life in terms of physical, psychosocial, and mental function.^[^ ^4^ ^]^ To date, various therapeutics have been used to manage CISDs with anti‐inflammatory and immune‐modulating effects: steroids, calcineurin inhibitors, methotrexate, cyclosporine, or biologics.^[^ ^5^ ^]^ Despite appropriate treatments, however, recalcitrant lichenified skin lesions persist (Figure 1a), demanding additional interventions.

In these cases, physicians inject triamcinolone acetonide (TA), an anti‐inflammatory drug, directly into skin lesions using needle‐syringe systems, namely, triamcinolone intralesional injection (TAILI) (Figure 1b). TAILI is widely administered and accepted for treating lichenified lesions of CISDs.^[^ ^6^ ^]^ Directly delivering TA to the dermis and lower epidermis by bypassing the thickened stratum corneum and epidermis (Figure 1a, right panels), the most potent barrier for drug delivery,^[^ ^7^ ^]^ enables TA to efficiently alleviate the pathologic features of CISDs.^[^ ^8^ ^]^ Despite its efficacy, the severe pain caused by TAILI causes patients to be stressed and reluctant to undergo treatment, especially children and elderly individuals.^[^ ^6^, ^9^ ^]^ Furthermore, due to the technical attributes of TAILI itself, the physician‐dependent amount and depth of the TA injection^[^ ^10^ ^]^ and uneven distribution of TA in the skin make it difficult to predict the outcome from lesion to lesion. Additionally, several side effects can occur, such as persistent scarring secondary to ulceration,^[^ ^11^ ^]^ linear atrophic streaks,^[^ ^12^ ^]^ or dyspigmentation in perilesional skin.^[^ ^13^ ^]^ In this respect, there is a tremendous medical need for a painless and standardized drug delivery system for evenly delivering TA into skin lesions, considering the high prevalence and substantial burden of CISDs globally.^[^ ^1^ ^]^

Herein, we thus propose a novel candle flame (“candlelit”)‐shaped dissolving microneedle (Candlelit‐DMN) conjoined with an applicator (Figure 1d), based on the application of microneedle products for the treatment of various skin diseases that are widely investigated, including infectious skin diseases, acne, and skin cancer.^[^ ^14^ ^]^ By taking advantage of the DMN,^[^ ^15^ ^]^ Candlelit‐DMN delivers TA in a minimally invasive manner without pain or biohazardous material. It can deliver a larger amount of drug into the lower layer of skin tissue than conventional cone‐shaped microneedles (Conv‐DMNs) (Figure 1c), and distribute drug evenly into skin tissue in a standardized manner, overcoming the disadvantages of TAILI. After confirming the drug delivery depth and distribution of Candlelit‐DMN inserted with the applicator, we validated the anti‐inflammatory pharmacodynamics in alleviating CISDs using a mouse model with skin inflammation in vivo.

## 2 Results

### 2.1 Design and Fabrication of Two Types of TA‐Loaded DMN

**Figure 2:**
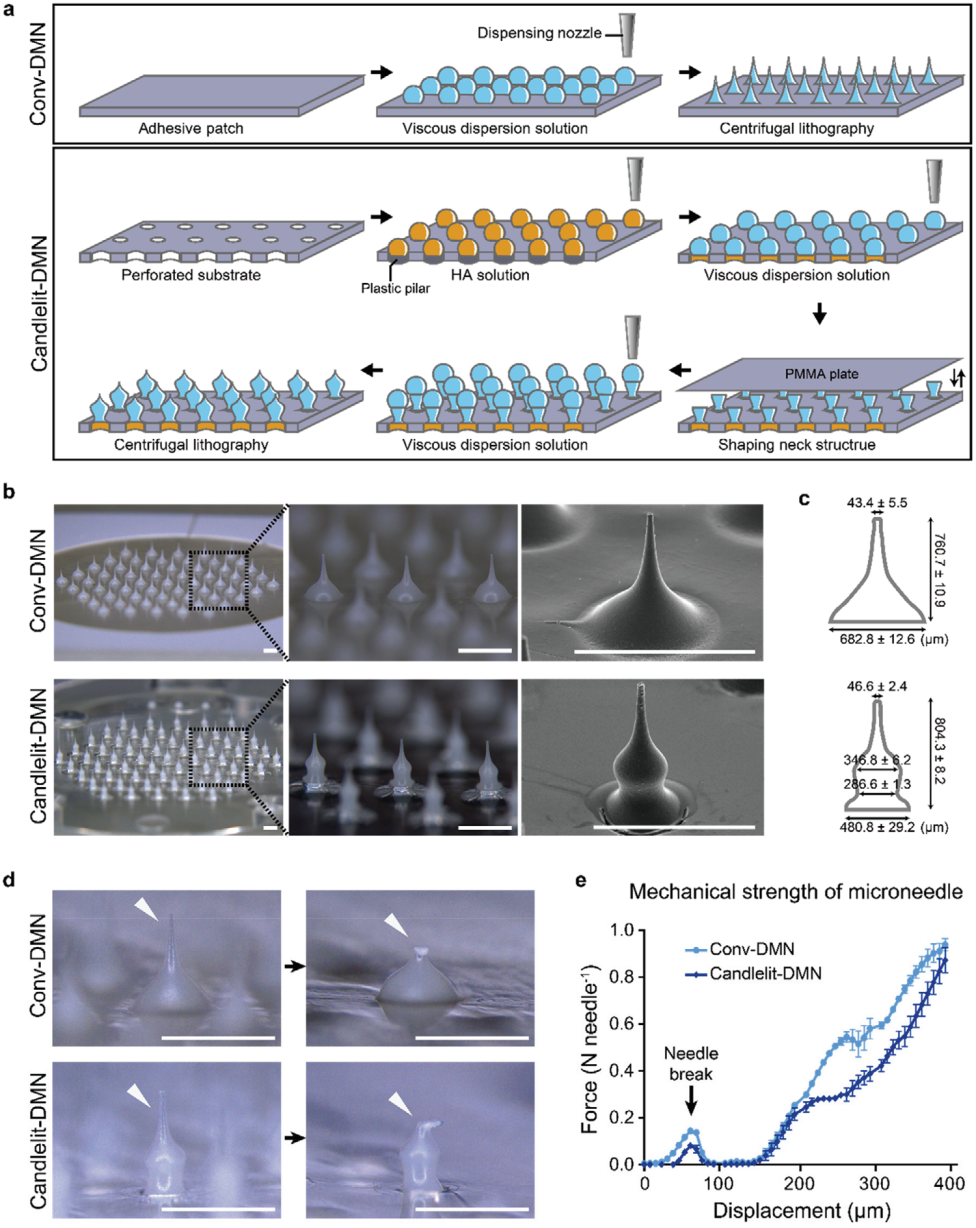
Fabrication and evaluation of the physical properties of Conv‐DMN and Candlelit‐DMN. a) Schematics of the fabrication method of Conv‐DMN (upper panel) and Candlelit‐DMN (lower panel). b) Microscopic and scanning electron microscopic images of Conv‐DMN (upper panel) and Candlelit‐DMN (lower panel). c) Geometric specifications of the Conv‐DMN and Candlelit‐DMN (mean ± s.e.m., *n* = 5 in each type). d) Microscopic image of the tip of the Conv‐DMN and Candlelit‐DMN (white arrowhead) before and after the evaluation of physical strength. e) Fracture force of Conv‐DMN and Candlelit‐DMN (*n* = 3 in each type). White bar: 1 mm.

For designing and fabricating TA‐loaded DMN, a viscous mixture consisting of TA, hyaluronic acid (HA), and polyvinylpyrrolidone (PVP) was prepared (Figure S1, Supporting Information) as a prerequisite for the DMN shape‐forming and drying process (**Figure** **2**a). As backbone molecules of DMN, we selected HA and PVP because these polymers are safe and FDA‐approved biocompatible materials.^[^ ^16^ ^]^ Using the viscous mixture, we fabricated Conv‐DMN in a streamlined, conical shape that narrows toward the ends (Figure 2a upper panel and Figure 2b upper panel).^[^ ^17^ ^]^ However, the fact that Conv‐DMN could deliver TA to the superficial rather than deep layer, as most TA was loaded in the base part (Figure 1c and Figure 2c upper panel),^[^ ^18^ ^]^ implied that Conv‐DMN could not reasonably mimic the TAILI procedure. To deliver more TA into the deeper skin, we fabricated Candlelit‐DMN (Figure 2a lower panel and Figure 2b lower panel). This DMN is hourglass‐shaped from the bottom of the structure to the middle height and then narrow from the middle to the tip,^[^ ^18^ ^]^ suggesting that Candlelit‐DMN would be superior to Conv‐DMN in that a greater portion of the DMN structure could reach the dermis layer (Figure 1d). The total TA doses loaded in the 61 DMN arrays (Figure 2b and Figure S2, Supporting Information) were 1.21 ± 0.03 mg (Conv‐DMN) and 1.20 ± 0.02 mg (Candlelit‐DMN) (mean ± s.e.m., *n* = 5, respectively), as a result of quantitative analysis, which was enough and comparable to the TAILI procedure.^[^ ^6^, ^8^ ^]^

### 2.2 Evaluation of Physical Properties of DMN

Next, we evaluated the physical properties of each DMN. The Conv‐DMN had a traditional conical shape, and the Candlelit‐DMN had a candlelight‐like shape with a neck in the middle (Figure 2b). The height of Conv‐DMN and Candlelit‐DMN were similar, showing 760.7 ± 10.9 and 804.3 ± 8.2 µm, which were comparable to the depths of the upper dermis and lower epidermis in lichenified lesions of CISDs (Figure 1a). The tip diameter of Conv‐DMN and Candlelit‐DMN were 43.4 ± 5.5 and 46.6 ± 2.4 µm, respectively, without any statistical significance (*p* = 0.8889). The base diameter of Conv‐DMN was 682.8 ± 12.6 µm, which narrowed toward the tip, whereas Candlelit‐DMN showed a candlelight‐like shape with a base diameter of 480.8 ± 29.2 µm, neck diameter of 286.6 ± 1.3 µm and head diameter of 346.8 ± 6.2 µm (Figure 2c). The relative volume distributions of Conv‐DMN and Candlelit‐DMN structures were calculated, and 86.8 ± 0.3% of each Conv‐DMN volume was distributed in the lower quarter, which was different from Candlelit‐DMN (53.6 ± 0.5% in the lower quarter) (Figure S3, Supporting Information). To evaluate whether the two types of DMNs have sufficient physical strength to penetrate the skin, we measured the fracture force of both DMNs (Figure 2d and Figure 2e). The fracture forces of Conv‐DMN and Candlelit‐DMN were 0.143 ± 0.006 N and 0.081 ± 0.005 N, respectively, which are sufficient to be inserted into the skin.^[^ ^19^ ^]^

### 2.3 Applicator for Candlelit‐DMN into Human Skin In Vivo

**Figure 3:**
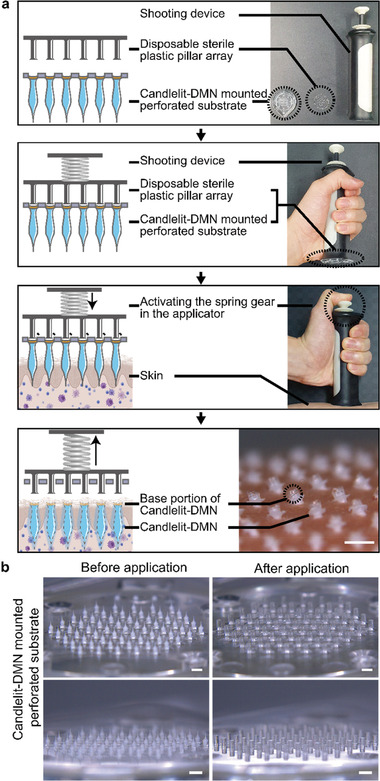
Applicator for applying Candlelit‐DMNs. a) The disposable pillar array and the Candlelit‐DMN mounted perforated substrate are set on the shooting device. Activating the spring gear in the shooting device causes each plastic pillar to hit each Candlelit‐DMN, resulting in each Candlelit‐DMN being stably inserted into the human skin in vivo. b) The perforated substrate on which Candlelit‐DMNs are mounted is observed with a microscope before and after activating the shooting device of the applicator. White bar: 1 mm.

**Figure 4:**
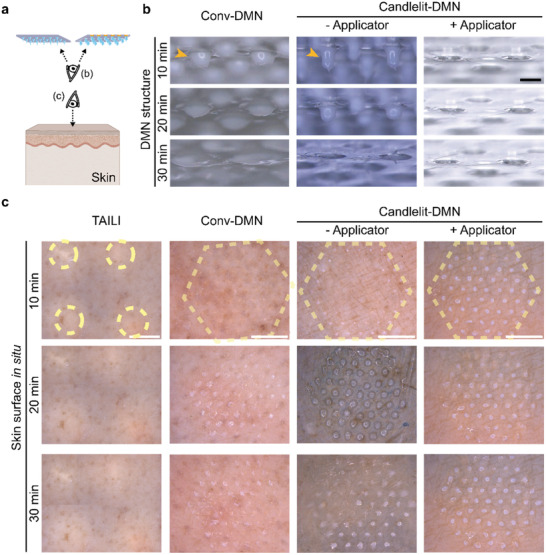
Drug delivery regularity of TAILI, Conv‐DMN, and Candlelit‐DMN using ex vivo human skin on the surface. a) Schematics of viewpoint for images in (b) and (c). b) Stereoscopic images of remnant DMN structures after applying Conv‐DMN and Candlelit‐DMN. (orange arrowhead: remnant DMN structure) c) Stereoscopic images of ex vivo human skin after TAILI or application of Conv‐DMN and Candlelit‐DMN (dotted yellow line: TA application site of each group) for 30 min at 10‐min intervals. Black bar: 500 µm; White bar: 5 mm.

Applying DMN structures by finger could result in interpersonal variation and not provide enough force, especially in lichenified lesions of CISDs.^[^ ^20^ ^]^ We developed an applicator to apply each Candlelit‐DMN into the skin in a uniform manner (**Figure** **3**a), which was composed of i) a shooting device, ii) a disposable sterile plastic pillar array, and iii) a Candlelit‐DMN mounted perforated substrate (Figure 3a, first panel). The disposable sterile plastic pillar array, which was designed to push each Candlelit‐DMN, could improve the quality of the insertion of Candlelit‐DMN into the skin by exerting uniform force onto each DMN mounted on the perforated substrate, in contrast to the DMN applied by finger, which is difficult to apply with uniform force. Furthermore, Candlelit‐DMN with an applicator system is expected to shorten the application process compared to the DMN on patch, which should be applied for more than 30 min (**Figure** **4**b). In addition, the disposable sterile plastic pillar array compartmentalizes the spaces of the shooting device and the perforated substrate, which prevents contamination of the applicator with body fluids or tissues and protects the patients from a potentially contaminated shooting device.

The applicator operated in the following order (Figure 3a and Movie S1, Supporting Information): i) The disposable pillar array and the Candlelit‐DMN mounted perforated substrate were set on the shooting device. ii) After placing the shooting device on the target skin, we activated the spring gear, causing each plastic pillar to hit each Candlelit‐DMN. As a result, iii) each Candlelit‐DMN was stably inserted into human skin in vivo. To confirm whether the Candlelit‐DMNs were consistently detached from the perforated substrate by the applicator, we observed the perforated substrate before and after using the applicator (Figure 3b). Before application, we observed that 61 Candlelit‐DMNs were mounted on the perforated substrate. After application, the 61 plastic pillar structures protruded from the perforated substrate through each hole, and all Candlelit‐DMNs were soundly separated and released from the perforated substrate (Figure 3b, Movie S1, Supporting Information). The released DMNs were stably inserted into human skin in vivo, leaving only the base portion (consisting of only HA without TA) exposed on the surface (Figure S4, Supporting Information). Collectively, the introduction of a disposable pillar array and the perforated substrate facilitated the insertion of each Candlelit‐DMN into the human skin in vivo. In addition, the plastic pillars were not inserted into the skin, suggesting no risk of damage or infection from the pillars.

### 2.4 Drug Delivery Regularity in Human Skin Ex Vivo

We fabricated two types of DMNs, Conv‐DMNs, and Candlelit‐DMNs, and developed an applicator for Candlelit‐DMNs. To evaluate the drug delivery performance according to the shape of the DMN and use of an applicator, we used ex vivo human skin, into which we performed TAILI and applied Conv‐DMN and Candlelit‐DMN with or without the applicator. Afterward, the skin surface and remnant structures of DMNs were observed for 30 min (Figure 4a). After DMN was applied to the skin and detached, some parts of the DMN structures of Conv‐DMN and Candlelit‐DMN without the applicator were left on the patch or the perforated substrate (Figure 4b), which were dissolved and creamed (Figure 4b). In contrast, the DMNs of the Candlelit‐DMN with the applicator group were completely separated, leaving no remnants (Figure 4b, third column).

On the skin surface, TAILI sites were noted as white spots, implying that TA molecules were observed through the skin. For 30 min, as expected in the current clinical practice, the white‐colored areas remained without any change (Figure 4c), meaning that the injected TA was not evenly distributed and the concentration gradient was steep across the skin. In contrast, the DMN structures were inserted with regularity into the skin, implying that the orderly arranged DMNs were suitable for evenly distributing TA into the skin (Figure 4c). However, it was observed that not all 61 DMNs of the Conv‐DMN and Candlelit‐DMN without applicator group were inserted uniformly, given that the dissolution degrees of the inserted DMNs were different among the DMNs for 30 min, leaving the creamed DMNs diffused on the surface (Figure 4c). In contrast, each creamed Candlelit‐DMN applied by the applicator notably occupied a smaller skin surface area than the other two groups. These results implied that the Candlelit‐DMN could be inserted uniformly and deeply using the applicator. Collectively, Candlelit‐DMN with the applicator was superior to Conv‐DMN and Candlelit‐DMN without the applicator in terms of drug delivery regularity.

### 2.5 Drug Delivery Depth and Distribution in Human Skin Ex Vivo

**Figure 5:**
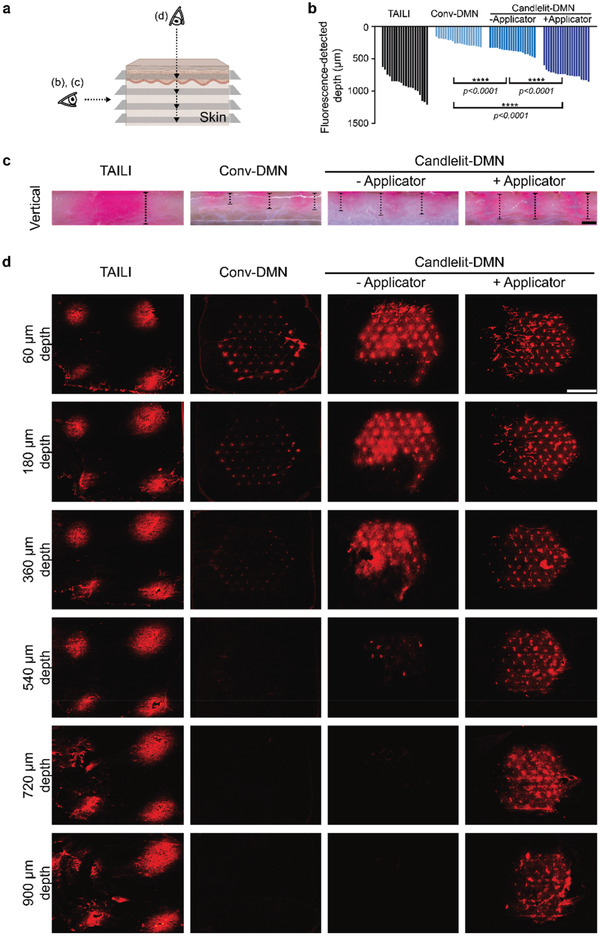
Drug delivery depth and distribution of TAILI, Conv‐DMN, and Candlelit‐DMN in ex vivo human skin. a) Schematics of viewpoint for images in (b), (c), and (d). b,c) The fluorescence depth is measured in the vertical section view after TAILI or application of Conv‐DMN and Candlelit‐DMN into the ex vivo human skin (*n* = 20 in each group). ****p < 0.0001; one‐way ANOVA with Tukey's multiple comparisons test. d) Each horizontally sectioned ex vivo human skin after TAILI or application of Conv‐DMN or Candlelit‐DMN was observed under a fluorescence microscope. Black bar: 500 µm, White bar: 5 mm

Now, we evaluated whether DMNs could reach the targeted skin layer: upper dermis and lower epidermis of lichenified lesions of CISDs (depth ranging 500 to 900 µm, Figure 1a). For this, a TA suspension with fluorescent dyes was prepared for TAILI and for Conv‐DMN and Candlelit‐DMN fabrication and administered to human skin, which was examined from vertical and transverse section views (**Figure** **5**a). The fluorescence‐detected depths were 916.2 ± 33.8 (TAILI), 238.8 ± 10.5 (Conv‐DMN), 384.2 ± 10.0 (Candlelit‐DMN without the applicator), and 749.9 ± 12.7 µm (Candlelit‐DMN with the applicator) (Figure 5b, c). The depth of Candlelit‐DMN with the applicator soundly achieved the target depth for lichenified lesion of CISDs and was significantly deeper than that of Conv‐DMN or Candlelit‐DMN without the applicator (*p* < 0.0001) (Figure 5b). The fluorescence‐detected depth when the Conv‐DMN was inserted using the applicator was also measured (Figure S5a, Supporting Information). Although the fluorescence‐detected depth of Conv‐DMN was increased by the applicator (Figure S5b, Supporting Information), the fluorescence‐detected depth of Conv‐DMN with applicator would not reach the target depth, and it was shallower than those of both the Candlelit‐DMN without and with the applicator (Figure 5b and Figure S5b, Supporting Information).

In terms of drug distribution in the skin, the dye of TAILI was focally localized in the injected spots and not transferred to the midst area (Figure 5d), corresponding to the skin surface (Figure 4c). From 60 to 900 µm depth, the dye was not detected between the injected spots, indicating that the injected TA was focalized, which could increase the possibility of drug side effects and an uneven treatment effect for each area (Figure 5d). In contrast, the dye of the DMN groups was widespread across the applied areas (Figure 5d). Specifically, compared to Conv‐DMN, Candlelit‐DMN was able to disperse more dye into the deep skin tissue (Figure 5d), given that the middle bulging portion of Candlelit‐DMN could aid in insertion into skin and delivery of more TA (Figure 2c). When Candlelit‐DMN was applied with the applicator, the dye was detected at up to 900 µm depth, and the 61 DMNs were more evenly inserted than those without the applicator (Figure 5d, third and fourth column). We confirmed that Candlelit‐DMN conjoined with the applicator could deliver TA into the deeper skin layer, mimicking the current TAILI procedure, and distribute TA evenly into the skin tissue in a standardized manner, overcoming the disadvantage of TAILI. Based on the aforementioned pharmacokinetics, whether Candlelit‐DMN with an applicator could provide ample anti‐inflammatory pharmacodynamics in vivo needed to be verified.

### 2.6 Anti‐Inflammatory Pharmacodynamics In Vivo

**Figure 6:**
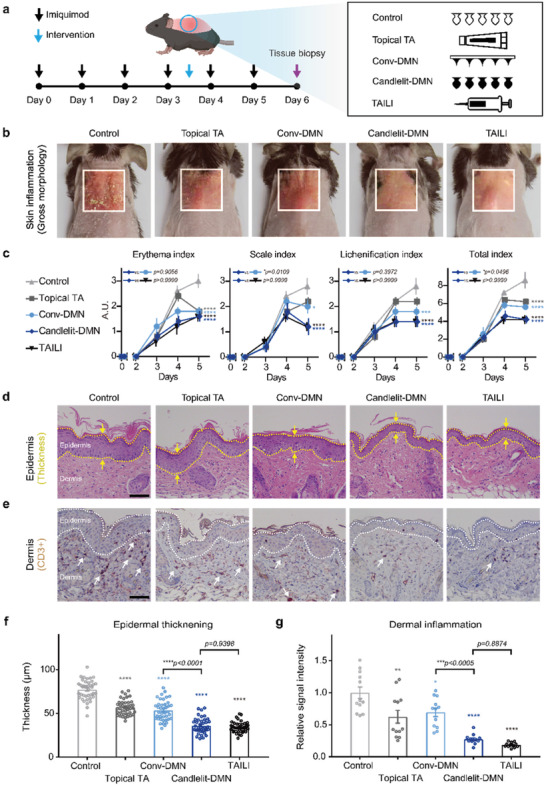
Anti‐inflammatory pharmacodynamics of Candlelit‐DMN with an applicator in vivo. a) Experiment timeline. b) The representative gross morphology of mice at day 6. c) The skin inflammation severity index (*n* = 5 in each group). *p = 0.0496, ***p = 0.0002, ****p < 0.0001 compared to the control group for each index on day 6; two‐way ANOVA with Tukey's multiple comparisons test. d) Representative histologic manifestations (H&E staining). Epidermis is in the yellow dotted line. The thickness of the epidermis was measured (yellow arrow). e) Representative immunohistochemical manifestations. Epidermis is in the white dotted line. The immunocytes in the dermis are indicated by white arrows. Black bar: 100 µm. f,g), Epidermal thickening and dermal inflammation of mouse skin tissue (*n* = 40 and *n* = 12 spots in each group, respectively). *p = 0.0204, **p = 0.0024, ****p < 0.0001 compared to the control group; one‐way ANOVA with Tukey's multiple comparisons test.

Before evaluating pharmacodynamics, we checked whether Candlelit‐DMN with the applicator could deliver TA properly in skin tissue in vivo. It was confirmed that TA was properly delivered in the skin tissue by the application of Candlelit‐DMN (Figure S6, Supporting Information), based on the fact that the concentration of TA reached its peak after 6 h of application, and then gradually decreased (Figure S6, Supporting Information). Next, to validate the anti‐inflammatory effects of Candlelit‐DMN with the applicator in vivo, we used a well‐established mouse model induced by topical imiquimod treatment,^[^ ^21^ ^]^ manifesting characteristics of CISDs, such as erythema, scale, and lichenification. After inducing dorsal skin inflammation with imiquimod, we intervened with the inflamed skin by applying TA topically, Conv‐DMN, Candlelit‐DMN with the applicator, and TAILI in each group (**Figure** **6**a). Topical TA treatment, one of the first‐line treatments for CISDs,^[^ ^5^ ^]^ was used as an indicator of the effectiveness of TA in this mouse model. The Candlelit‐DMN was applied only with the applicator based on the aforementioned confirmed results: the Candlelit‐DMN conjoined with the applicator could deliver TA into the skin tissue more efficiently than that without the applicator.

We quantified skin inflammation daily in terms of erythema, scale, and lichenification (Figure 6b and Figure 6c). The erythema index was significantly lower in all the TA‐treated groups than in the control group (*p*<0.0001), implying that TA in each modality effectively modulated inflammation. With respect to the scale index, the Candlelit‐DMN with the applicator and TAILI groups (1.20 ± 0.75 and 1.20 ± 0.40, respectively) showed significantly lower scores than the control group (2.80 ± 0.75) (*p* < 0.0001). The mice in the Conv‐DMN group also showed a lower scale index than the control group (2.00 ± 0.63, *p* = 0.0109). Notably, the antiscale effect of Candlelit‐DMN with the applicator was comparable to that of TAILI and more effective than that of Conv‐DMN (*p* = 0.0109). Similar results were found for the lichenification index. The Candlelit‐DMN with the applicator and TAILI groups had similarly low lichenification index scores compared to the control group (1.40 ± 0.49 in both group vs 2.80 ± 0.75 in control; *p* < 0.0001). Overall, mice in the groups treated with TA showed fewer inflammatory features than those in the control group (*p* < 0.0001) (Figure 6b and Figure 6c fourth panel). Focusing on Candlelit‐DMN with the applicator, we found that its anti‐inflammatory effect was superior to that of Conv‐DMN (*p* = 0.0496) and comparable to that of TAILI (Figure 6b).

Thickening of the skin epidermis is also an indicator of inflammation.^[^ ^21^ ^]^ We measured epidermal thickness (Figure 6d) and found that it was significantly reduced in all TA‐treated groups compared with the control group (*p* < 0.0001) (Figure 6f). Among the treatments, Candlelit‐DMN with the applicator efficiently reduced the epidermal thickness compared to Conv‐DMN and topical TA (35.95 ± 1.41 µm vs 53.44 ± 1.83 µm or 57.01 ± 1.37 µm, *p* < 0.0001, respectively). Remarkably, the epidermal thinning effect of Candlelit‐DMN with the applicator was comparable to that of TAILI (35.95 ± 1.41 µm vs 34.32 ± 0.98 µm, *p* = 0.9398) (Figure 6f). We also checked T cells in the dermis as an indicator of inflammation severity,^[^ ^22^ ^]^ based on the signal intensity of CD3 in immunohistochemically stained skin tissue (Figure 6e). Consistent with the epidermal thickening results, Candlelit‐DMN with the applicator more competently reduced inflammation than Conv‐DMN (*p* < 0.0005) (Figure 6g). Its effect was comparable to that of TAILI (*p* = 0.8874) (Figure 6g). Collectively, the anti‐inflammatory effect of Candlelit‐DMN with the applicator was comparable to that of TAILI and superior to that of Conv‐DMN.

### 2.7 Inflammatory Gene Expression Modulating Effects In Vivo

We also evaluated inflammatory gene expression levels in each group in an in vivo model. As imiquimod‐induced skin inflammation is related to genes such as interleukin (IL)‐1B, IL‐6, IL‐12, IL‐17A, IL‐23, and tumor necrosis factor‐alpha (TNF*α*),^[^ ^23^ ^]^ we measured the mRNA levels of these genes using quantitative reverse transcription‐polymerase chain reaction (RT‐qPCR).

**Figure 7:**
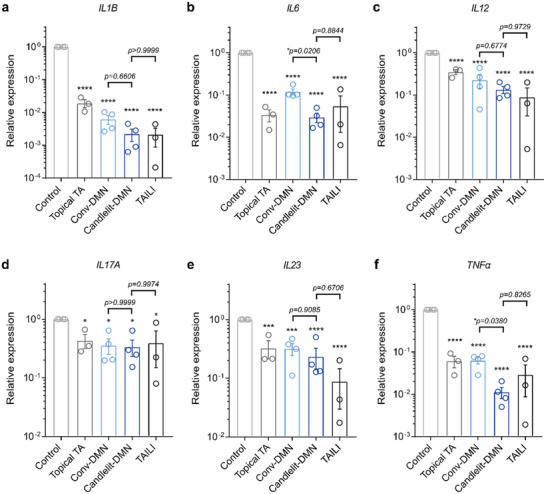
Modulating inflammatory cytokine gene expression in vivo. a–f) The relative gene expression levels of each inflammatory cytokine gene (*n* = 3 or 4 in each group). *p < 0.05, ***p < 0.001, ****p < 0.0001 compared to the control group for each gene; one‐way ANOVA with Tukey's multiple comparisons test.

We found that the expression levels of all investigated genes were significantly decreased in the four TA‐treated groups compared with the control group (**Figure** **7**), consistent with the gross morphology results (Figure 6). Compared to the Conv‐DMN group, the Candlelit‐DMN with the applicator group showed greater potency in reducing gene expression, especially IL‐6 and TNF*α* (*p* = 0.0206 and *p* = 0.0380, respectively) (Figure 7b,f). These results implied that Candlelit‐DMN with the applicator was superior in alleviating inflammation, considering that IL‐6 is essential for the development of epidermal thickening^[^ ^24^ ^]^ and antagonizing TNF*α* is an established treatment for CISD.^[^ ^25^ ^]^ Overall, Candlelit‐DMN with the applicator was comparable to TAILI in regard to reducing inflammatory gene expression levels.

### 2.8 Preference for Candlelit‐DMN with Applicator

Thus far, we had validated the pharmacokinetics and pharmacodynamics of Candlelit‐DMN with the applicator. We next determined whether it could be used in a real‐world clinical setting similar to the current TAILI procedure. The video (Movie S1, Supporting Information) and the photo taken after insertion of the microneedle into human skin (Figure S4, Supporting Information) were presented to dermatologists (*n* = 24; 13 board‐certified dermatologists and 11 dermatology residents). Overall, dermatologists were interested in Candlelit‐DMN with the applicator as a standardized method with even delivery of TA and DMNs that are dissolved after application, leaving no waste, in contrast to the needle‐syringe system that generates biohazardous sharps waste. When asked which method they preferred as a method for treating lichenified lesions of CISDs, the dermatologists indicated a greater preference for the Candlelit‐DMN with the applicator than for the current TAILI using needle‐syringe system (Figure S7, Supporting Information).

## 3 Discussion

The skin is the largest organ in the human body, functioning as the outermost interface for physical and social interaction with the external environment. Impairing its integrity not only causes diseases but also negatively impacts well‐being and self‐esteem,^[^ ^26^ ^]^ leading to depression and suicide,^[^ ^27^ ^]^ CISDs is the most prominent cause of lasting and disfiguring changes in the skin^[^ ^26^ ^]^ due to its chronic and relapsing features. Along this line, CISD is not just a simple disease and demands active management and treatment. To manage CISDs, TAILI is one of the most widely used medical procedures to treat recalcitrant lichenified lesions.^[^ ^6b,c^ ^]^ However, the TAILI procedure needs to be improved and standardized because this procedure is painful, delivers the drug unevenly, and depends on practitioners’ maneuvers, resulting in interpractioner variation.

Mechanical nociception of the skin involves damage to nociceptive sensory nerve endings that can occur after physical insult to the skin.^[^ ^28^ ^]^ Because the sensory nerve endings in the skin diverge like a tree branch structure, perpendicular insertion of needles can minimize pain by intersecting fewer nerves.^[^ ^29^ ^]^ The Candlelit‐DMNs are inserted into the skin more perpendicular to the skin surface than the needle in TAILI. Besides the needle insertion direction, the volume or insertion rate of structures inserted into the skin tissue are significant factors in evoking pain.^[^ ^10^, ^30^ ^]^ In the current TAILI procedure, a 0.1–0.2 mL TA suspension is injected at each point (1 cm^2^).^[^ ^6c^ ^]^ In contrast, the total volume of our Candlelit‐DMN inserted per 1 cm^2^ was only 1.93×10^−3 ^mL (out of 61 needles, 55 were located within 1 cm^2^ with each needle volume being 0.035 ± 0.001 mm^3^; Figure 2 and Figure S2, Supporting Information), which is much smaller than the volume used in the TAILI procedure. Considering that minute volume insertion is crucial in reducing pain,^[^ ^10^, ^30^ ^]^ our Candlelit‐DMN could induce less pain than the TAILI procedure. Indeed, DMN application proved to be not painful and was well tolerated and accepted in humans.^[^ ^31^ ^]^ Collectively, adopting a DMN structure for delivering TA into the skin is reasonable in terms of reducing pain.

Compared to TAILI, Candlelit‐DMN could evenly distribute the drug across the applied area, which would be important for reducing the likelihood of side effects that occur due to the presence of high volume or/and concentrations of localized TA in the skin. Despite its wide usage in intralesional injection, TA itself is more likely to produce atrophy in the skin than other drugs.^[^ ^32^ ^]^ Skin ulceration due to blockage of the blood microcirculation by slough^[^ ^6^, ^30^ ^]^ and cutaneous calcification caused by precipitation of chalky white material at the injection site^[^ ^6a^ ^]^ would occur.

The main purpose of the TAILI procedure in treating CISDs is to inject TA into the upper dermis and lower epidermis, where TA affects dermal infiltrative immunocytes, as well as keratinocytes of the epidermis.^[^ ^4^, ^6^, ^33^ ^]^ Along this line, we designed the Candlelit‐DMN, which can deliver more TA into deeper layers of skin than a Conv‐DMN, reasonably mimicking TAILI. As shown in this study, Candlelit‐DMNs could deliver TA into the deeper skin layer. This result could be attributed to the improved insertion efficiency of the Candlelit‐DMN shape, in which the diameter increases from the tip to the middle part and then decreases to the bottom of the Candlelit‐DMN, reducing the resistance during the insertion process. In addition to the drug delivery efficacy, the geometry with sharp tips, wide bodies, and narrow neck structures could enhance skin embedding with interlocking effects.^[^ ^18^ ^]^ Indeed, the ratio of the length of the microneedle that is not inserted into the skin to the total length was significantly lower in a Candlelit‐DMN than in a Conv‐DMN.^[^ ^18^ ^]^

The TAILI procedure necessarily causes variations in the amount and depth of injected drug,^[^ ^10^ ^]^ which would interfere with predicting prognosis to the treatment. The manual application of DMNs, such as fingers, also cannot be free from this drawback. Equipped with the applicator, Candlelit‐DMNs could provide standardized treatment with reduced variation among healthcare providers in terms of injecting a fixed dose of TA with a specific depth at every injection.

## 4 Conclusion

In short, we started by addressing the unmet medical needs of the current TAILI procedure in treating CISDs. To evenly deliver TA into the skin without pain, we adopted the DMN structure, followed by examining the histopathology of CISDs to incorporate the optimal design of the DMN to mimic TAILI: Candlelit‐DMN. We devised its applicator for standardized application, which would also force Candlelit‐DMN to be inserted into lichenified skin. The Candlelit‐DMN with the applicator was effective in a mouse model. We believe that this could be a plausible candidate fulfilling the needs of both patients and practitioners, tackling the disadvantages of current TAILI with needle‐syringe systems and thereby improving patient adherence to treatment. Furthermore, it might be possible for patients to apply DMNs without trained medical staff after proper guidance, accommodating patient convenience and reducing medical costs considering the high cost of TAILI.^[^ ^34^ ^]^

We believe that the Candlelit‐DMN with an applicator would be more profitable than the current TAILI procedure; nonetheless, clinical trials in humans should be preceded to assess both safety and efficacy before it is used in actual clinical practice, considering that 1) no clinical data regarding the effects of the insertion of the DMN structure on human skin integrity in vivo; 2) no experimental data availability using severely lichenified human skin tissue in vivo; and 3) the possibility that the applicator would not function properly on highly curved skin surfaces.

Furthermore, considering that previous clinical studies have demonstrated that TAILI is effective for various diseases, such as alopecia areata, vitiligo, keloid, hypertrophic scar, nail disorders, and acne,^[^ ^35^ ^]^ this TA delivery system could be used for such disorders. At the time, the volume of Candlelit‐DMN should be optimized to adjust the mounted TA dose for each disease, and it should be noted that the insertion efficiency of the DMN may change depending on the head diameter of the Candlelit‐DMN. Moreover, this drug delivery system could be used for drugs that are intralesionally injected into the skin, such as botulinum toxin or anticancer drugs,^[^ ^36^ ^]^ in the future.

## 5 Experimental Section

### Study Design

This study was designed with the objective of inventing a Candlelit‐DMN with an applicator, which could be used as the current TAILI procedure for treating humans affected with CISDs. The approach strategy was first to design a Candlelit‐DMN satisfying the following criteria: 1) use of Food and Drug Administration approved materials and drug molecules; 2) a sharp tip strong enough to penetrate the epidermis and upper dermis of skin tissue; 3) load of TA dose compatible with human therapeutic effects; 4) a high concentration of TA in the DMNs to minimize the inserted volume to reduce pain; 5) a purposefully designed DMN to optimize drug delivery targeting the dermis layer; 6) a conjoined standardized and simple application for even distribution of TA; and 7) no biohazard waste remaining. The developed Candlelit‐DMN with the applicator was studied in an in vivo CISD mouse model to validate its ability to satisfy these needs.

### Fabrication of Conv‐DMN and Candlelit‐DMN

To fabricate Conv‐DMN (Figure 2a upper panel), TA (1.13 g, Sigma‐Aldrich, USA) was premixed in deionized water (5 mL) and sonicated it using an ultrasonic cleaner (WiseClean WUC, Witeg, Germany) for 30 min.^[^ ^37^ ^]^ PVP (0.25 g, Jiaozuo Zhongwei Special Products Pharmaceutical Co., China) and HA (2.25 g, Bloomage Freda Biopharm, China) were added to the premixed TA solution and mixed using a paste mixer (PDM‐300C, KM TECH Co. Ltd., Korea) for 30 min, resulting in the viscous dispersion solution (Figure S1, Supporting Information). The viscous solution was dispensed onto an adhesive patch, resulting in 61 arrays using a dispenser (ML‐5000X, Musashi Engineering, Inc., Japan) (Figure S2, Supporting Information). Afterward, Conv‐DMN was formed by using the centrifugal lithography technique (Figure 2a upper panel).^[^ ^38^ ^]^

For fabricating Candlelit‐DMN (Figure 2a lower panel), a perforated substrate made of general purpose polystyrene (GPPS) material was first prepared, in which 61 holes of 400 µm in diameter were arranged with 1.5 mm intervals in a hexagonal shape (Figure S2, Supporting Information). Next, the holes were closed to a depth of 100 µm by using 350 µm‐diameter plastic pillars (made with GPPS) arranged in the same manner as the perforated substrate. Afterward, the base solution (2.50 g of HA was added to 5 mL of deionized water and mixed for 30 min using the paste mixer) was dispensed into each hole of the perforated substrate and dried for 3 h, resulting in a base structure. The viscous solution, consisting of TA (22.5%, w/v), HA (45.0%, w/v), and PVP (5%, w/v) in deionized water, was dispensed onto the base structure, and the poly(methyl methacrylate) (PMMA) plate was attached to fabricate the neck structure at 400 µm intervals with a perforated substrate and dried for 3 h. After removing the PMMA plate, the viscous solution was dispensed onto the neck structure. Afterward, the final Candlelit‐DMN was formed by using the centrifugal lithography technique (Figure 2a lower panel).^[^ ^38^ ^]^

Conv‐DMN and Candlelit‐DMN with a fluorescent dye were prepared by mixing 0.1% (w/v) rhodamine B (Sigma‐Aldrich, USA) in deionized water (5 mL), and then the same manufacturing process was used to make Conv‐DMN and Candlelit‐DMN. Also, for the application of the Conv‐DMN with applicator, it was fabricated using a viscous solution mixed with 0.1% (w/v) rhodamine B. The viscous solution was dispensed and formed Conv‐DMN by using centrifugal lithography on a perforated substrate with a base structure.

### Evaluation of the Shape and Physical Strength of Conv‐DMN and Candlelit‐DMN

Images of Conv‐DMN and Candlelit‐DMN were acquired using a fluorescent stereomicroscope (M165FC, Leica Camera AG, Wetzlar, Germany) and a software (LAS Version 4.12.0 [Build: 86], Leica Microsystems, Heerbrugg, Switzerland). A scanning electron microscope (SEM, JEOL‐7610F‐Plus, JEOL Ltd., Tokyo, Japan) was used to obtain images of Conv‐DMN and Candlelit‐DMN. The fracture force was measured using a material testing machine (OmniTest 5.0, Mecmesin Ltd, West Sussex, United) and a software (VectorPro 5.2.0.0., Mecmesin Ltd, West Sussex, United Kingdom). A single Conv‐DMN or Candlelit‐DMN was fixed to the test stage of the material testing machine, and the test probe was moved downward at a continuous speed of 3.6 mm min^−1^. After the probe reached the tip of the single microneedle, the axial force when the Conv‐DMN or Candlelit‐DMN fractured was recorded as the fracture force.

### Analysis of Average Volume Distribution Comparison of Conv‐DMN and Candlelit‐DMN

Analysis of average volume distribution comparison of Conv‐DMN and Candlelit‐DMN was performed using software (Autodesk Inventor Professional 2021, Autodesk, Inc., San Rafael, CA, USA). Three DMN images were loaded from each of the experimental groups and calculated the volume of each DMN using the software. The volume of the DMN was calculated by dividing it into quarters based on the total length of each DMN structure.

### Quantification of TA Loaded on Conv‐DMN and Candlelit‐DMN

Quantitative analysis of Conv‐DMN and Candlelit‐DMN was performed using high‐performance liquid chromatography (HPLC, Waters Alliance 2695 Separations Module, Waters 996 PDA detector with Empower 2 software, Milford, MA, USA) according to the monograph of the Korean Pharmacopoeia.^[^ ^39^ ^]^ In brief, prednisolone (10 mg, Sigma‐Aldrich, St. Louis, MO, USA) was dissolved in methanol (50 mL, Sigma‐Aldrich, St. Louis, MO, USA) to prepare the internal standard (I. STD) solution. The mobile phase was prepared by degassing a mixture of water and acetonitrile (3:1, v:v, Sigma‐Aldrich, St. Louis, MO, USA). Test solution was prepared by mixing methanol, I. STD solution, and the mobile phase in a 1:1:3 ratio to make a total solution (10 mL) and Conv‐DMN and Candlelit‐DMN in this solution. A standard solution was prepared by dissolving TA standard (20 mg, Sigma‐Aldrich, St. Louis, MO, USA) in methanol to make an exact total solution (50 mL) and this solution (10 mL), I. STD solution, and the mobile phase in a 1:1:3 ratio, respectively. TA quantities in Conv‐DMN and Candlelit‐DMN were calculated by the following equations:

(1) $$\begin{array}{ccc} & & \mathrm{TA}\;\mathrm{content}\;\mathrm{in}\;\mathrm{DMN}\;\left(\mu g\right)=\mathrm{TA}\;\mathrm{content}\;\mathrm{in}\;\mathrm{TA}\;\mathrm{standard}\;\mathrm{solution}\;\left(\mu g\right)\\ & & \times \;Qt/Qs\times \mathrm{Dilution}\;\mathrm{factor}\;\left(10\right)\\ & & *Qt=\mathrm{TA}\;\mathrm{peak}\;\mathrm{height}/\mathrm{Prednisolone}\;\mathrm{peak}\;\mathrm{height}\;\mathrm{in}\;\mathrm{test}\;\mathrm{solution}\\ & & *Qs=\mathrm{TA}\;\mathrm{peak}\;\mathrm{height}/\mathrm{Prednisolone}\;\mathrm{peak}\;\mathrm{height}\;\mathrm{in}\;\mathrm{standard}\;\mathrm{solution} \end{array}$$

### Conception of the Applicator System for Candlelit‐DMN

By refining a previously presented shooting device,^[^ ^40^ ^]^ an applicator system composed of a shooting device, a disposable sterile plastic pillar array, and a Candlelit‐DMN mounted perforated substrate was devised (Figure 3a). This applicator system for Candlelit‐DMN is i) a custom‐made shooting device powered by a spring gear with a tension of 3.6 kgf, ii) a custom‐made 1.5 mm long, 350 µm diameter, 61 pillar structure made of GPPS arranged in a hexagonal shape with 1.5 mm intervals, and iii) a custom‐made perforated substrate made of GPPS material mounted with 61 Candlelit‐DMNs on each hole with a diameter of 400 µm, arranged in a hexagonal shape at 1.5 mm intervals (Figure 3b).

### Evaluation of the Ex Vivo Skin Human Surface after TAILI and Application of Conv‐DMN or Candlelit‐DMN

Human cadaver skin (HansBiomed Corp., Korea) was thawed for 30 min at room temperature in phosphate‐buffered saline (PBS) buffer solution (Sigma‐Aldrich, USA), and moisture was removed from the surface of human cadaver skin using a wipe. TA (40 mg mL^−1^) (Triam inj., Shin Poong Pharmaceuticals, Korea) was diluted using saline solution (3 mg mL^−1^) and injected (0.1 mL doses) at 1 cm intervals into the prepared human cadaver skin. After injection, 4 to 5 images were obtained from the skin, according to the position using a stereomicroscope, and the images were merged to the actual position to represent the entire application area as one image (Figure S8, Supporting Information).^[^ ^41^ ^]^ Conv‐DMN or Candlelit‐DMN was applied to the skin using the thumb, and their supporting materials (patch or perforated substrate) were removed after 30 min. For the Candlelit‐DMN with the applicator group, the DMNs were applied with the applicator system. The DMN structures and skin were photographed using a stereomicroscope every 10 min for 30 min.

Evaluation of dye delivery depth and distribution within ex vivo human skin tissue after TAILI and applying Conv‐DMN or Candlelit‐DMN: TA (3 mg mL^−1^) with 0.1% rhodamine B (w/v) was injected (0.1 mL doses) at 1 cm intervals into the prepared human cadaver skin. Conv‐DMN and Candlelit‐DMN loaded with TA and fluorescent dye were applied to the skin using the thumb or applicator system. The DMNs were placed in situ into the skin tissue at room temperature for 10 min, and then the skin tissue was frozen at −20 °C for 3 h. Afterwards, the frozen skin was cut vertically with a razor. The cut section was observed using a fluorescent stereomicroscope, and the depth was measured from the skin surface to the deepest dyed part of each TA‐injected spot or DMN‐inserted spot.

For the horizontal sectioned view, TAILI, Conv‐DMN, and Candlelit‐DMN with fluorescent dye were applied to the prepared human cadaver skin. TA (3 mg mL^−1^) with rhodamine B (0.1% w/v) was injected (0.1 mL doses) at 1 cm intervals into the prepared human cadaver skin. Treatments in the Conv‐DMN and Candlelit‐DMN without applicator groups were applied using the thumb to human cadaver skin, and supporting materials (patch or perforated substrate) were removed after 30 min. For the Candlelit‐DMN with the applicator group, the DMNs were applied with the applicator into the skin. Afterward, the skin tissues were left at room temperature for 3 h after application, which is sufficient for the inserted DMN to dissolve.^[^ ^42^ ^]^ The skin tissues were embedded in OCT compound and horizontally sectioned at a thickness of 60 µm using a cryotome (CM 1850, Leica, Wetzlar, Germany). The sections were observed using a fluorescence microscope.

### Evaluation of Dye Delivery Depth of Conv‐DMNs With and Without an Applicator

Pig cadaver skin (CRONEX, Korea) was thawed for 30 min at room temperature in PBS solution, and moisture was removed from the surface of the skin using a wipe. Conv‐DMN with 0.1% (w/v) rhodamine B was applied to pig cadaver skin for 10 min using hand and the applicator. After that, the patch was removed, and the Conv‐DMN remaining on the skin surface was wiped off with a tissue. The applied skin tissue was frozen for 3 h at −20 °C, and the frozen skin was cut vertically with a razor blade. The drug delivery depth of the drug was analyzed by measuring the maximum depth of the dye using a fluorescent stereomicroscope.

### Evaluation of TA Concentration in In Vivo Rat Skin after Application of Candlelit‐DMN with Applicator

All experiments were performed in accordance with the protocol and guidelines approved by the Institutional Animal Care and Use Committee of Korea Conformity Laboratories. Eight‐week‐old male Crl:CD (Sprague Dawley) rats were purchased (Orient Bio Inc., Korea). The rats were maintained in the good laboratory practice (GLP) facility of Korea Conformity Laboratories in accordance with the Guide for the Care and Use of Laboratory Animals 8th edition, NRC (2010). After a week of acclimation, the rats were shaved and depilated, taking care not to damage the skin (*n*  =  7).

Candlelit‐DMN was applied with the applicator system in the back skin of rats. After a certain period of time after application (6 h, 1 d, 7 d, and 14 d), the central skin tissue of the Candlelit‐DMN‐applied site was harvested using an 8 mm disposable biopsy punch (Kai Industries Co., Ltd, Japan), washed thrice with sterile physiological saline, and then washed thrice with 70% alcohol. After weighing the skin tissue, the skin tissue was immersed in 50% methanol for 6 h, pulverized with a homogenizer, centrifuged at 3000 rpm for 30 min, and only the supernatant was used for analysis. Ten milligrams of TA (Sigma Aldrich, USA) was weighed and mixed in acetonitrile (J.T. Baker, USA) at a concentration of 200.0 mg L^−1^ to prepare the I. STD solution. Ten milligrams of dexamethasone (Sigma Aldrich, USA) was weighed and mixed in acetonitrile at a concentration of 5.0 mg L^−1^ to prepare a working solution. A total of 50 µL of 5.0 mg L^−1^ I. STD solution, 30 µL of 1 m sodium hydroxide (Sigma Aldrich, USA), and 800 µL of tert‐butyl methyl ether (Sigma Aldrich, USA) were added to 200 µL of a biological sample, followed by vortex mixing for 30 s. Then, it was centrifuged at 3400 rpm for 5 min to obtain 600 µL of the supernatant. After adding 90 µL of 0.1% formic acid (Sigma Aldrich, USA) to 600 µL of the supernatant, vortex mixing was performed for 30 s, and then centrifuged at 3400 rpm for 5 min to obtain 70 µL of the lower layer. The quantification value was obtained from the chromatogram, and the peak area ratio (peak area of the standard substance/peak area of the internal standard substance) of the standard substance to the peak area of I. STD was obtained. The concentration of the test substance was calculated from the calibration curve prepared in advance.

### Inducing and Quantifying Skin Inflammation in Mice

All experiments were performed in accordance with the protocol and guidelines approved by the Institutional Animal Care and Use Committee of Seoul National University Hospital. Seven‐week‐old female C57BL/6 mice were purchased (Koatech, Korea). The mice were maintained in the semi‐SPF facility accredited by AAALAC International (#001 169) in accordance with Guide for the Care and Use of Laboratory Animals 8th edition, NRC (2010). After a week of acclimation, the mice were randomly grouped into five experimental groups (*n*  =  5 in each group), and their backs were shaved and depilated, taking care not to damage the skin. Two days later (designated day 0), 5% imiquimod cream (62.5 mg for each mouse, Aldara cream, Dong‐A ST, Korea) was applied to the backs of the mice for six consecutive days to induce skin inflammation.^[^ ^21^ ^]^ To quantify the severity of inflammation of the skin, erythema, scale, and lichenification were scored independently by two blinded evaluators (J.O. and B.M.K.) every day using a scale system from 0 to 4 (0, none; 1, slight; 2, moderate; 3, marked; 4, very marked) for each, resulting in a total score by adding up three items (range: 0–12).

### Intervention by Topical TA, Conv‐DMN Candlelit‐DMN, or TAILI in Inflammation‐Induced Mouse Skin

One hour after imiquimod treatment on day 3, inflammation‐induced mice in each group were treated with i) Candlelit‐DMN without TA (designated the control group), ii) TA in cream formulation 1 g (Tricort Cream, Dongkwang Pharmaceuticals; concentration 0.1% w:w; designated the topical TA group), iii) Conv‐DMN, iv) Candlelit‐DMN, or v) TAILI (100 µL for 4 points; Triam inj, Shin Poong Pharmaceuticals; concentration 2.5 mg mL^−1^ in PBS).

### Mouse Tissue Preparation for Histological Analysis and RT‐qPCR

On day 6, the mice were sacrificed to obtain dorsal skin tissue (intervention area). The tissue was fixed in 4% neutral–buffered formaldehyde solution for 24 h and processed for paraffin embedding. Sections of 5 µm thickness were stained with hematoxylin & eosin and immunohistochemically stained using anti‐CD3 antibody (#ab237721, Abcam, USA). To quantify the epidermal thickness, the distance between the granular layer and basal layer of the epidermis was measured at ten spots in hematoxylin & eosin‐stained skin tissue of each mouse.

Total RNA was isolated from dorsal skin tissue using RNA iso Plus (Takara Bio Inc., Japan) and treated with DNase I (Roche Pharmaceuticals, Switzerland). One microgram of total RNA was used for the cDNA synthesis reaction, which was performed using a RevertAid First Strand cDNA Synthesis Kit (Thermo Scientific, USA). To quantify the mRNA expression of each gene, PCR was performed on a 7500 Real‐Time PCR System (Applied Biosystems, USA) using SYBR Premix Ex Taq (Takara Bio Inc., Japan). Sequences for the PCR primers are listed in Table S1, Supporting Information.

### Statistical Analysis

All of the results presented in this study were the means ± s.e.m. Statistical analysis was performed using one‐ or two‐way analysis of variance (ANOVA) with Tukey's multiple comparisons test, unpaired t test, or Mann‐Whitney test with Prism 8 software (GraphPad). Two‐sided *p* < 0.05 was considered statistically significant.

## Conflict of Interest

M.J., H.Y., and J.T.H. are employees of JUVIC, Inc. H.J. is an inventor of patents that have been licensed to companies developing microneedle products and is a founder/shareholder of companies developing microneedle products. This potential conflict of interest has been disclosed and is managed by Yonsei University.

## Supporting information

Supporting Information

Supporting Movie 1

## Data Availability

The data that support the findings of this study are available from the corresponding author upon reasonable request.
