# Supplementary material for: Dissolving Candlelit Microneedle for Chronic Inflammatory Skin Diseases
Source: Adv Sci (Weinh). 2021 May 7;8(14):2004873. doi: 10.1002/advs.202004873 (PMC8292898; doi:10.1002/advs.202004873)
Supplement: Supplementary file 1 — Supporting Information [file ADVS-8-2004873-s001.pdf]

© 2021 Wiley-VCH GmbH

ADVANCED SCIENCE
Open Access

Supporting Information

for *Adv. Sci.*, DOI: 10.1002/advs.202004873

Dissolving Candlelit Microneedle for Chronic Inflammatory Skin Diseases

Jungyoon Ohn, Mingyu Jang, Bo Mi Kang, Huisuk Yang, Jin Tae Hong, Kyu Han Kim, Ohsang Kwon, Hyungil Jung**

Supporting Information

Dissolving Candlelit Microneedle for Chronic Inflammatory Skin Diseases

Jungyoon Ohn, Mingyu Jang, Bo Mi Kang, Huisuk Yang, Jin Tae Hong, Kyu Han Kim, Ohsang Kwon, Hyungil Jung**

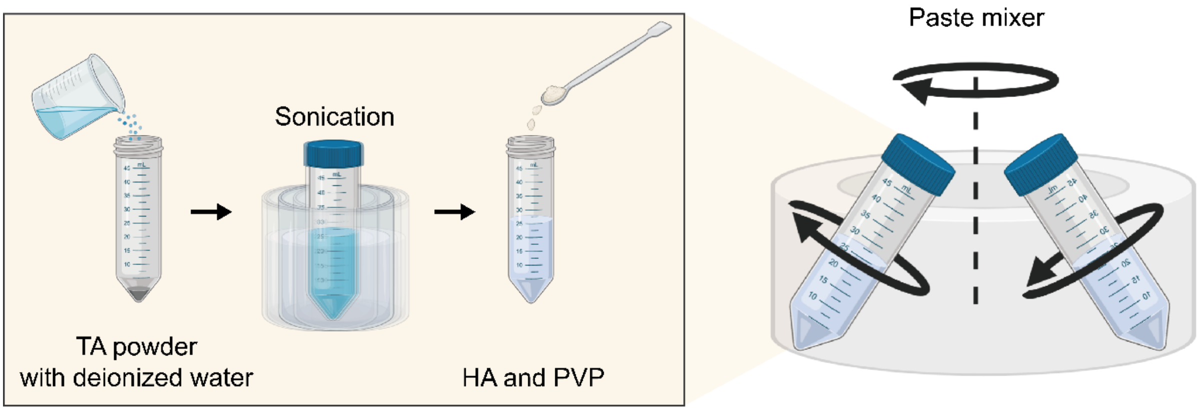

Figure S1. Preparing a mixture for fabricating DMNs, which consists of TA, HA, and PVP. After sonicating TA suspension, HA and PVP are added as backbone polymer. The paste mixer is used for the intensive mixing and the homogeneous dispersing of the viscous mixture.

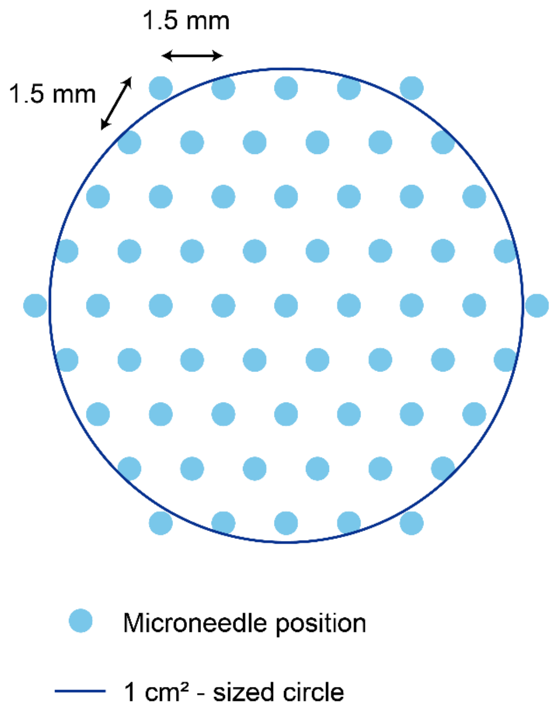

Figure S2. Schematic diagram of the fabricated microneedle array. A total of 61 microneedles were positioned (indicated as blue dots) at 1.5 mm intervals.

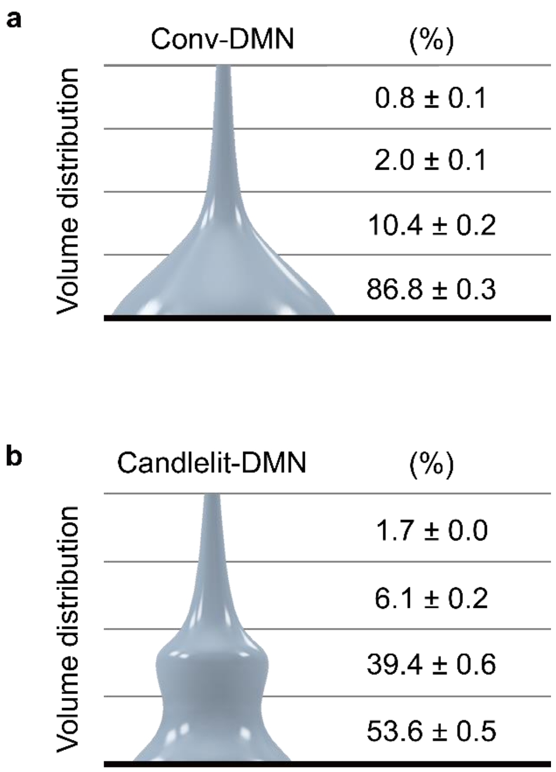

Figure S3. Relative volume distribution of each a) Conv-DMN and b) Candlelit-DMN (mean ± s.e.m, n = 3 in each group).

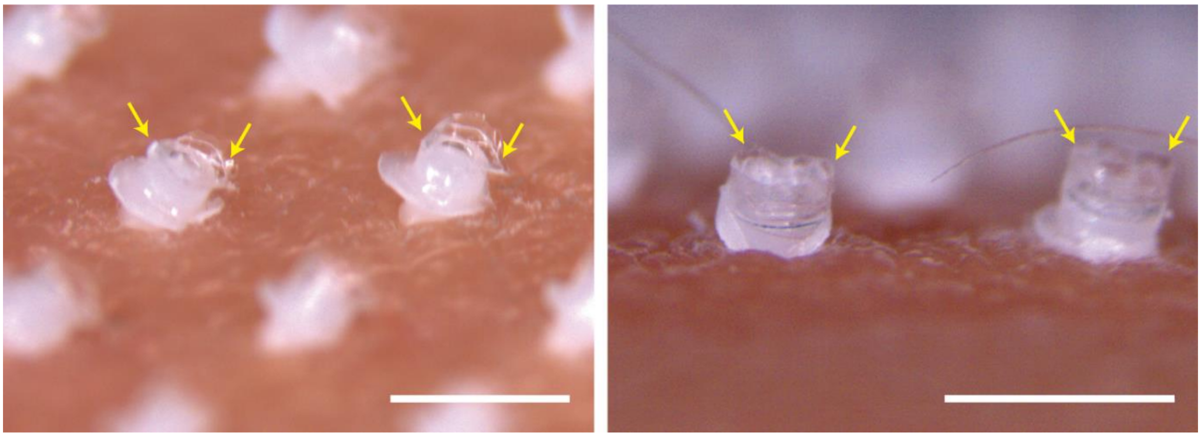

Figure S4. Stereoscopic image of Candlelit-DMNs in *in vivo* human skin. The consistently separated Candlelit-DMNs are evenly inserted into human skin *in vivo* in a stable manner, leaving only the base portion (yellow arrow), which consists of only HA without TA, on the skin surface. White bar: 1 mm.

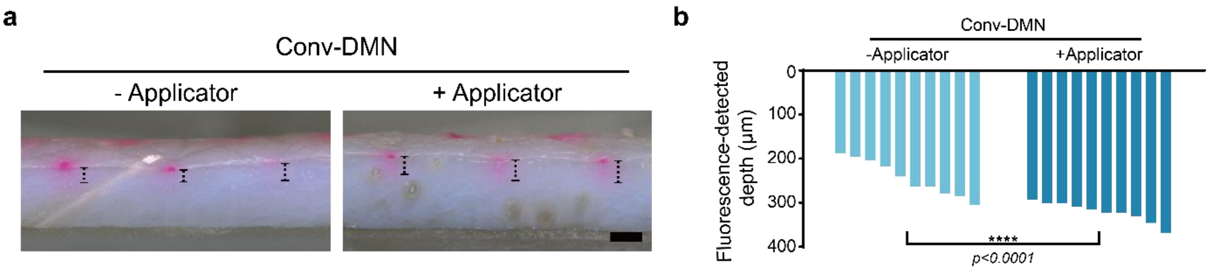

Figure S5. Comparison of the fluorescence-detected depth of Conv-DMN. a) Cross-section image after Conv-DMN application with and without applicator. Black bar: 500 µm. b) The fluorescence-detected depth was measured in the vertical section view after application of Conv-DMN with and without applicator use within ex vivo pig cadaver skin (*n* = 10 in each group). ****p < 0.0001; unpaired t test.

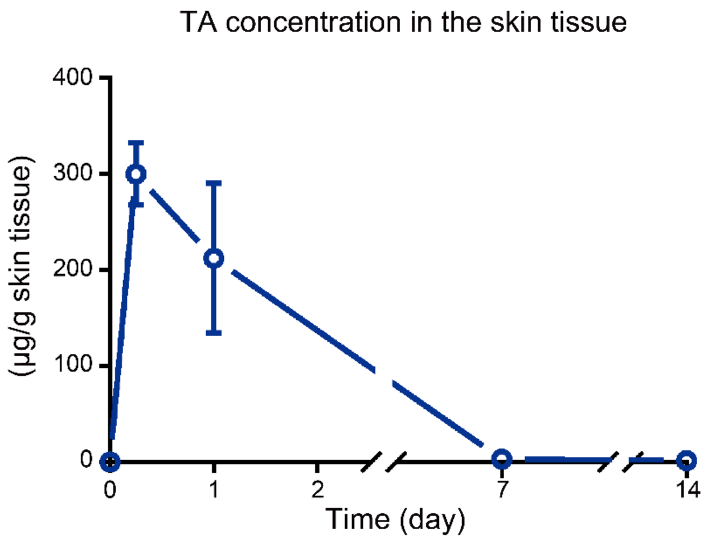

Figure S6. TA concentration in the skin tissue *in vivo* after application of Candlelit-DMN with applicator ($n = 7$).

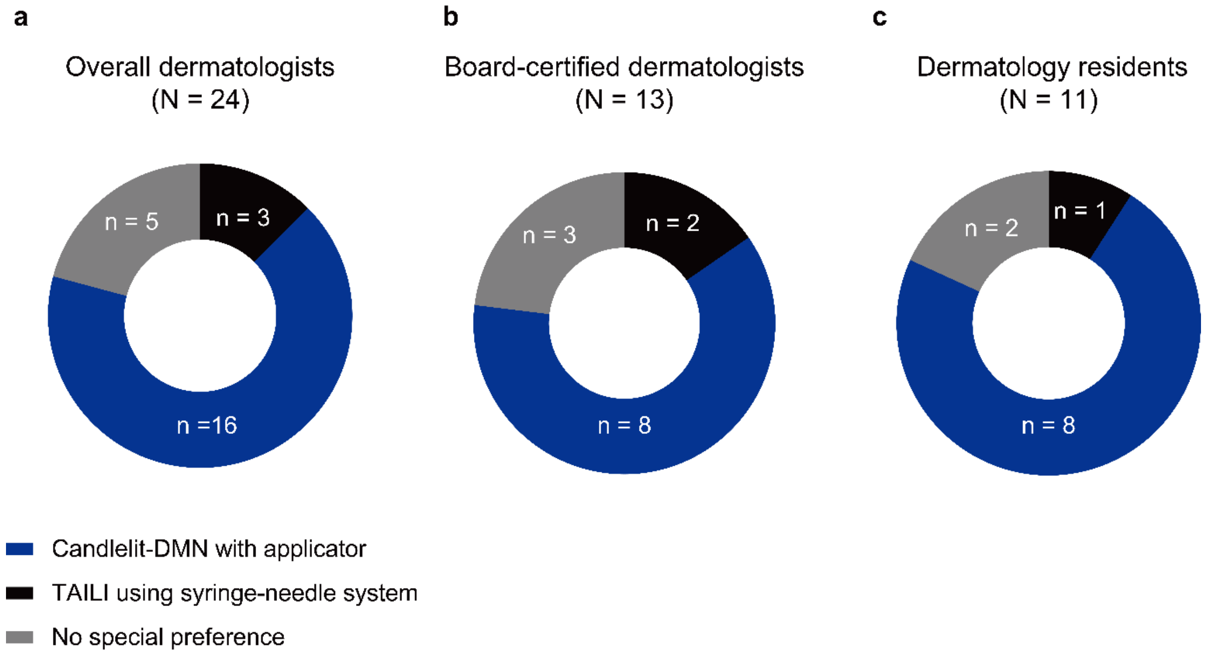

Figure S7. Preference for Candlelit-DMN with applicator over TAILI. a) among overall dermatologists, b) among board-certified dermatologist, c) among dermatology residents.

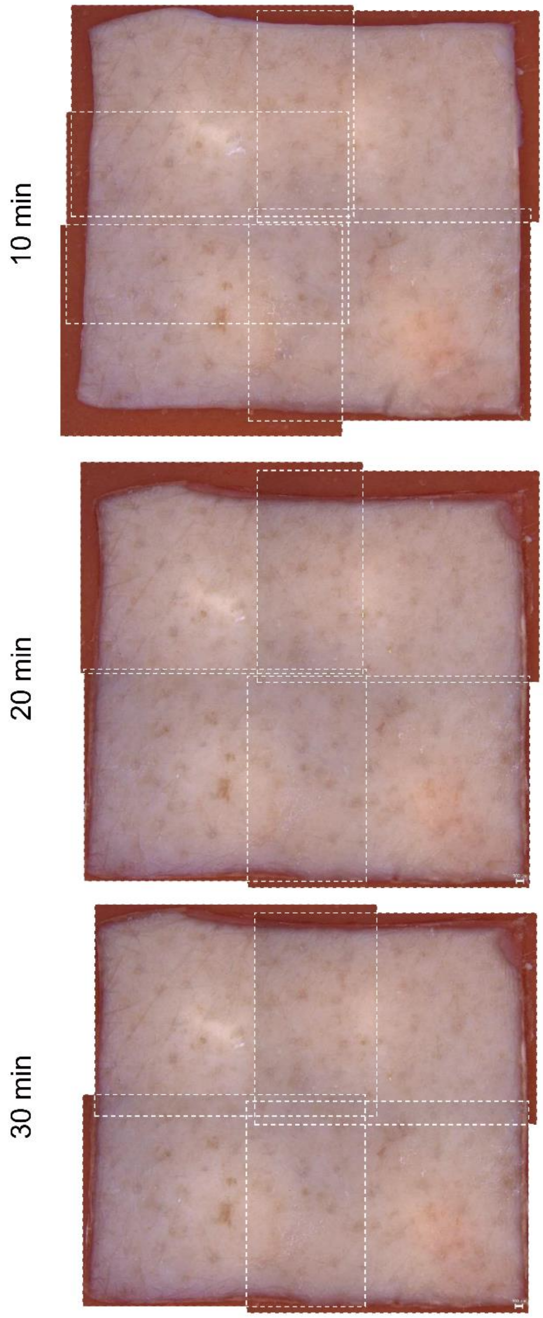

Figure S8. Stereoscopic image of human cadaver skin after TAILI. Each image is obtained for skin surface after TAILI at 10 minute intervals for 30 minutes.

Table S1. Sequences for the PCR primers

Mouse IL-1B	
sense	TGT AAT GAA AGA CGG CAC ACC
antisense	TCT TCT TTG GGT ATT GCT TGG
Mouse IL-17A	
sense	ATC CCT CAA AGC TCA GCG TGT C
antisense	GGG TCT TCA TTG CGG TGG AGA G
Mouse TNFα	
sense	TCT TCT CAT TCC TGC TTG TGG
antisense	GGT CTG GGC CAT AGA ACT GA
Mouse IL-6	
sense	CTT CCA TCC AGT TGC CTT CTT G
antisense	AAT TAA GCC TCC GAC TTG TGA AG
Mouse IL-12B	
sense	GGA AGC ACG GCA GCA GAA TAA
antisense	CTT GAG GGA GAA GTA GGA ATG
Mouse IL-23p19	
sense	CCA GCA GCT CTC TCG GAA TC
antisense	TCA TAT GTC CCG CTG GTG C
Mouse GAPDH	
sense	ACTGGCATGGCCTTCCGT
antisense	CCACCCTGTTGCTGTAGCC

Movie S1. Candlelit-DMN insertion with the applicator into human skin *in vivo*
